# Supplementary material for: An Optimized Workflow for the Analysis of Metabolic Fluxes in Cancer Spheroids Using Seahorse Technology
Source: Cells. 2022 Mar 2;11(5):866. doi: 10.3390/cells11050866 (PMC8909358; doi:10.3390/cells11050866)
Supplement: Supplementary file 1 [file cells-11-00866-s001.zip › Campioni et al - Supplementary materials_Revised 02-03-22.pdf]

cells MDPI

SUPPLEMENTARY MATERIALS

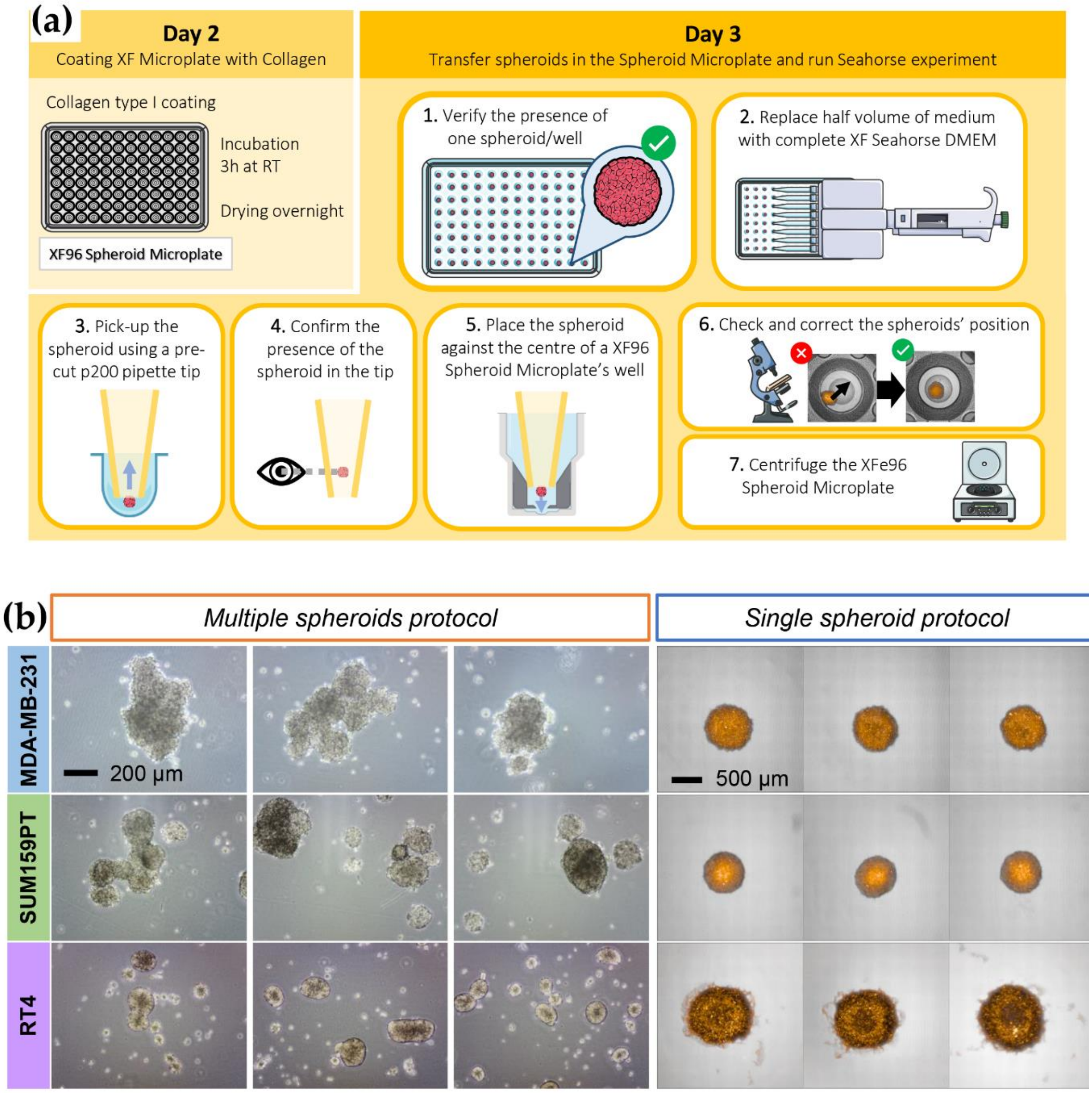

Figure S1. Protocol for spheroid transfer in XF Seahorse Microplate and different outcome for spheroid culture methods. **(a)** Schematic representation of spheroids' transfer in the XF Seahorse Microplate. **(b)** Representative pictures of MDA-MB-231 cell line, SUM159PT cell line (mammary carcinoma), and RT4 cell line (urothelial carcinoma) spheroids produced with the *Multiple spheroids protocol* (left) and the *Single spheroid protocol* (right) before their transfer in the XF Seahorse Microplate. Images were acquired in phase-contrast light microscopy (*Multiple spheroids protocol*) and brightfield and confocal fluorescent microscopy (in orange, CellTracker™ Red CMTPX Dye) with Operetta CLS™ (*Single spheroid protocol*).

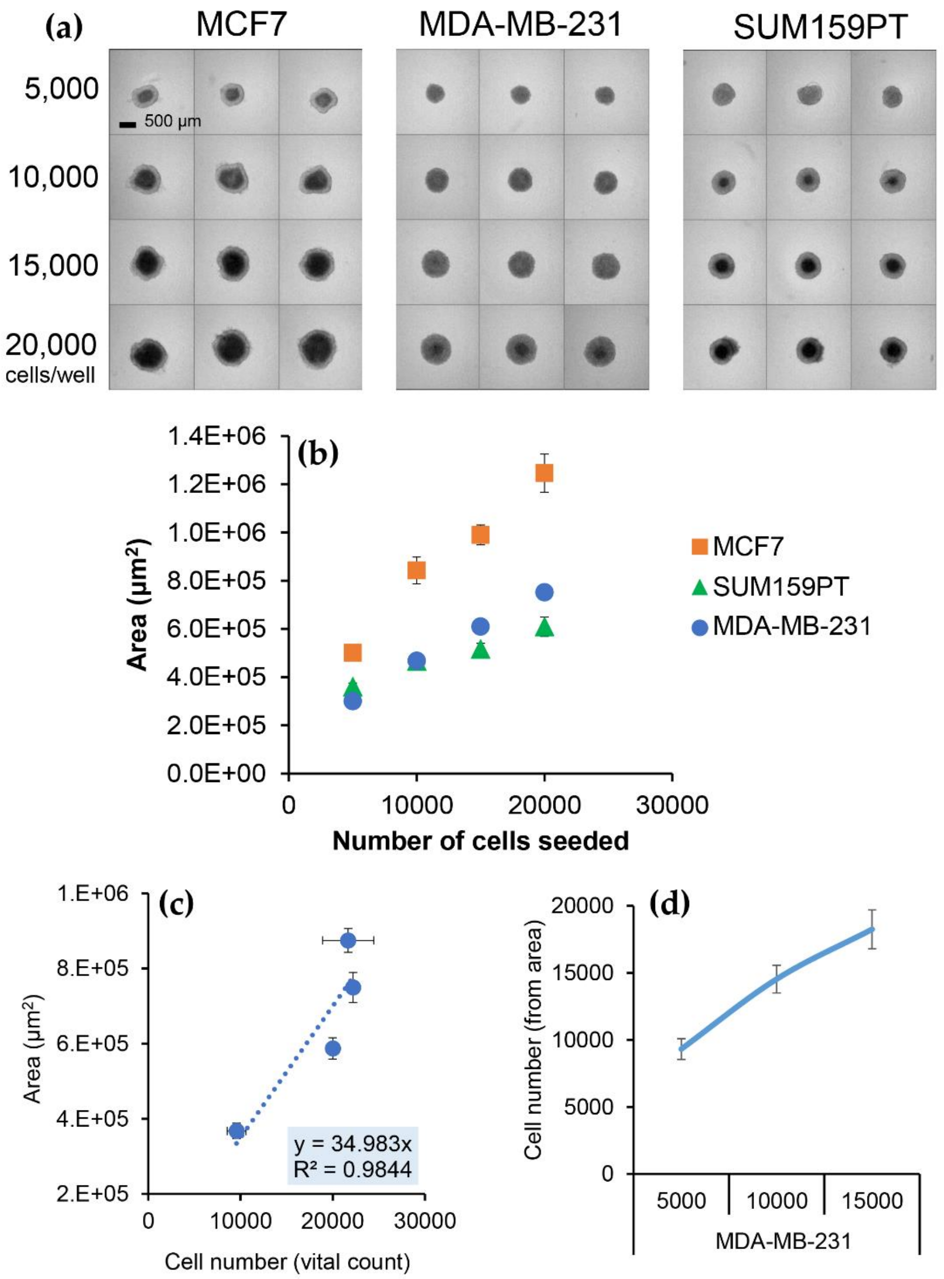

Figure S2. Impact of seeded cell number on morphology and dimension of spheroids obtained with the *Single spheroid protocol*. (**a**) Brightfield imaging with Operetta CLS™ of MCF7, MDA-MB-231, and SUM159PT spheroids produced by seeding a different number of cells/well: 5000 cells, 10000 cells, 15000 cells, and 20000 cells. (**b**) Correlation of the number of seeded cells and respective spheroid area obtained after 72 hours measured through imaging with Operetta CLS™ and analysis with Harmony software; points represent mean± standard deviation. (**c**) Standard curve obtained measuring the area of groups of MDA-MB-231 spheroids produced from 5,000 – 10,000 – 15,000 - 20,000 cells/well and their subsequent digestion and count of the number of vital cells after 72h of spheroid formation (note that the number of cells-per-spheroids after 72h of culture is different than the starting number of cells/well seeded). Points represent mean ± standard deviation along with regression. (**d**) Graph representing the number of cells-per-spheroid indirectly calculated using area measurement on MDA-MB-231 spheroids tested in a Seahorse assay, grouped based on the number of cells seeded for spheroids formation (5,000 – 10,000 – 15,000 cells/well). The points of the blue line depict the number of cells-per-spheroid calculated by measuring the area of each spheroid of the XF Seahorse Microplate and applying the equations derived from the standard curve in panel (**c**).

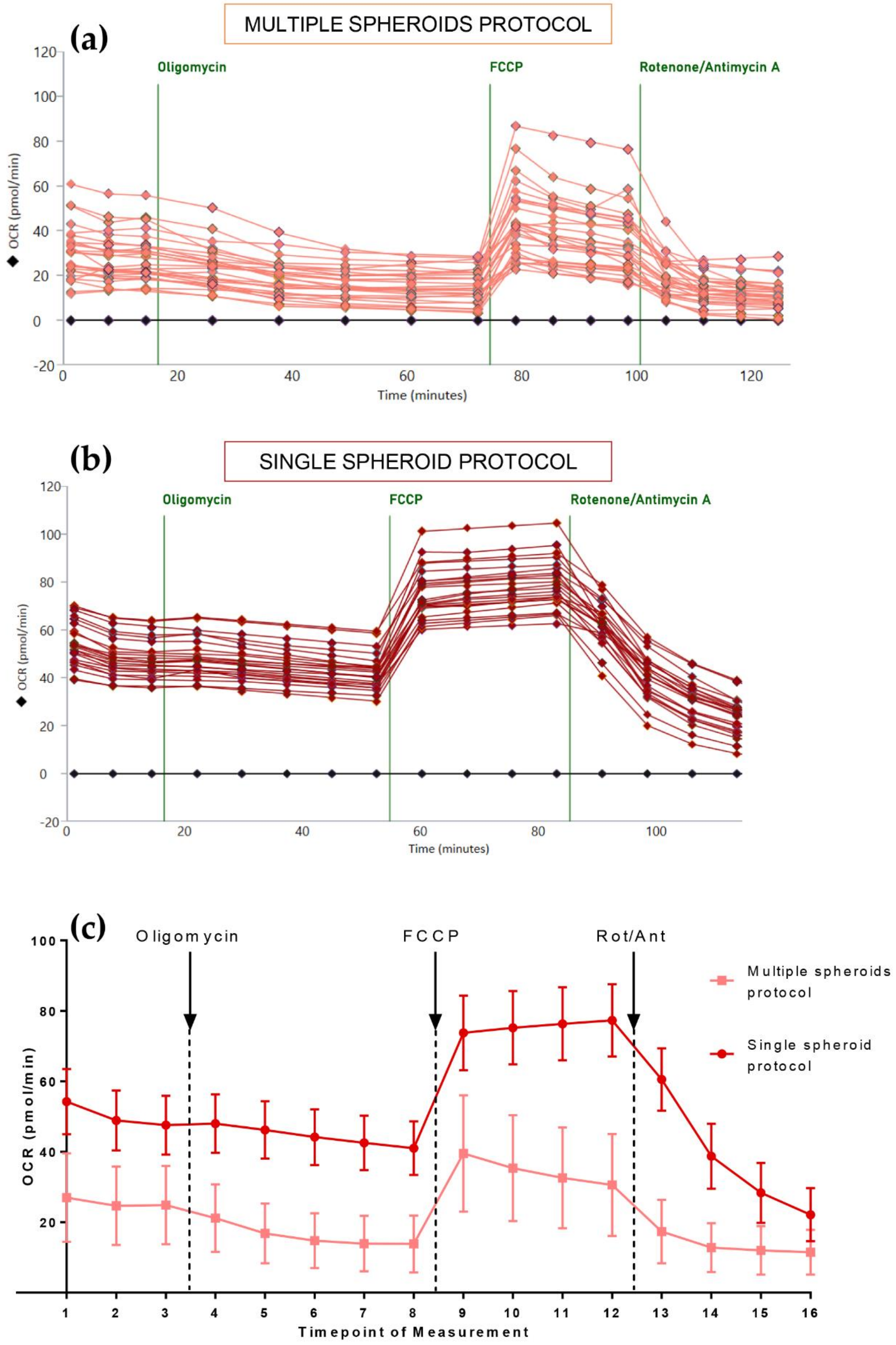

Figure S3. Comparison between *Multiple spheroids protocol* and *Single spheroid protocol* on MCF7 Mito Stress test profiles. OCR Profiles of Mito stress test produced with *Multiple spheroids protocol* (**a**) and *Single spheroid protocol* (**b**); single measurement for every spheroid is reported to show distribution, the black line is for background and corresponds to zero value. (**c**) The mean± standard deviation of OCR values is plotted for each measurement of the Mito stress test comparing the two protocols of spheroid formation.

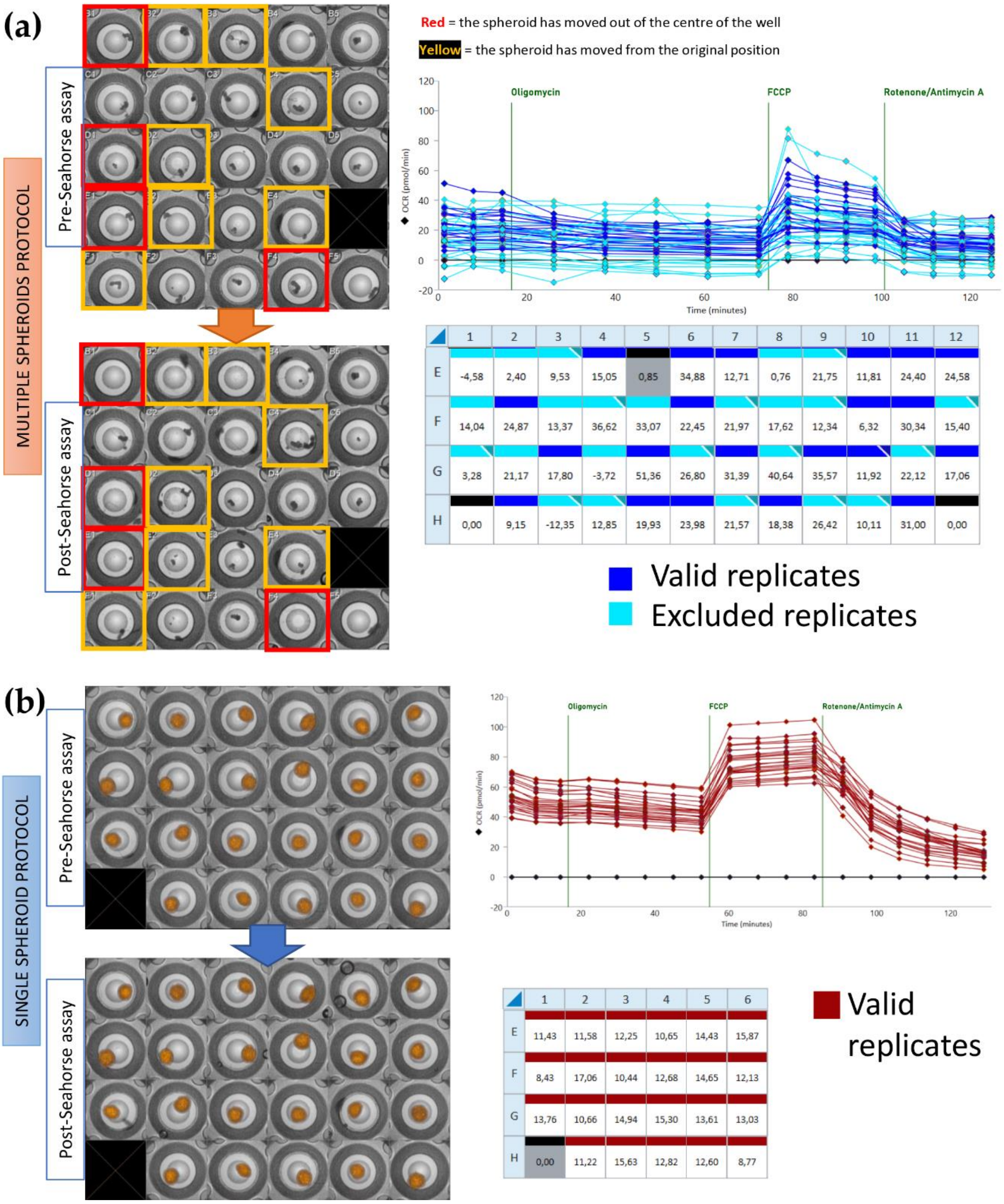

	1	2	3	4	5	6	7	8	9	10	11	12
E	-4,58	2,40	9,53	15,05	0,85	34,88	12,71	0,76	21,75	11,81	24,40	24,58
F	14,04	24,87	13,37	36,62	33,07	22,45	21,97	17,62	12,34	6,32	30,34	15,40
G	3,28	21,17	17,80	-3,72	51,36	26,80	31,39	40,64	35,57	11,92	22,12	17,06
H	0,00	9,15	-12,35	12,85	19,93	23,98	21,57	18,38	26,42	10,11	31,00	0,00

	1	2	3	4	5	6
E	11,43	11,58	12,25	10,65	14,43	15,87
F	8,43	17,06	10,44	12,68	14,65	12,13
G	13,76	10,66	14,94	15,30	13,61	13,03
H	0,00	11,22	15,63	12,82	12,60	8,77

Figure S4. Comparison of success rate in obtaining valid data with Seahorse technology between *Multiple spheroids protocol* and *Single spheroid protocol*. Representation through imaging (brightfield imaging with Operetta CLS™), Mito stress test profile plot, and plate schematic depiction of the success rate in obtaining valid data analyzing 3D structure with Seahorse technology choosing between *Multiple spheroids protocol* and *Single spheroid protocol*. (**a**) The *Multiple spheroids protocol* requires a strict selection of the replicates to eliminate those spheroids that have moved during the assay. The image shows examples of replicates that have been excluded from the analysis (light blue) and examples of replicates that have been conserved for the analysis (dark blue); red and yellow squares represent changes in the sample contained in the well, respectively movement out of the center of the well or from the original position. (**b**) All the replicates can be considered valid with the *Single spheroid protocol* since none of the spheroids have moved during the assay.

(a)

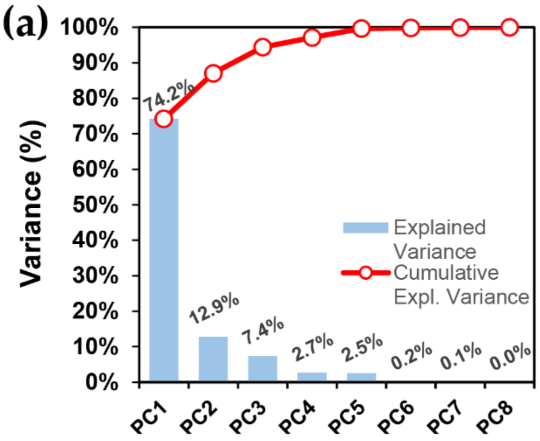

(b) Extracted Eigenvectors

Parameters	PC1	PC2
OCR baseline	**0.31134**	-0.11149
OCR oligomycin	**0.29918**	-0.1768
OCR FCCP	**0.29232**	**0.22906**
OCR Rot/Ant	0.16171	**-0.37051**
ECAR baseline	**0.30751**	-0.12045
ECAR oligomycin	**0.30831**	-0.08847
ECAR FCCP	**0.30911**	-0.05879
ECAR Rot/Ant	**0.30164**	-0.09284
Basal respiration	**0.3082**	0.06431
Maximal respiration	**0.26409**	**0.43785**
Spare capacity	0.01487	**0.71934**
ATP linked respiration	**0.27025**	0.13418
Proton leak	**0.29634**	0.02916

(c)

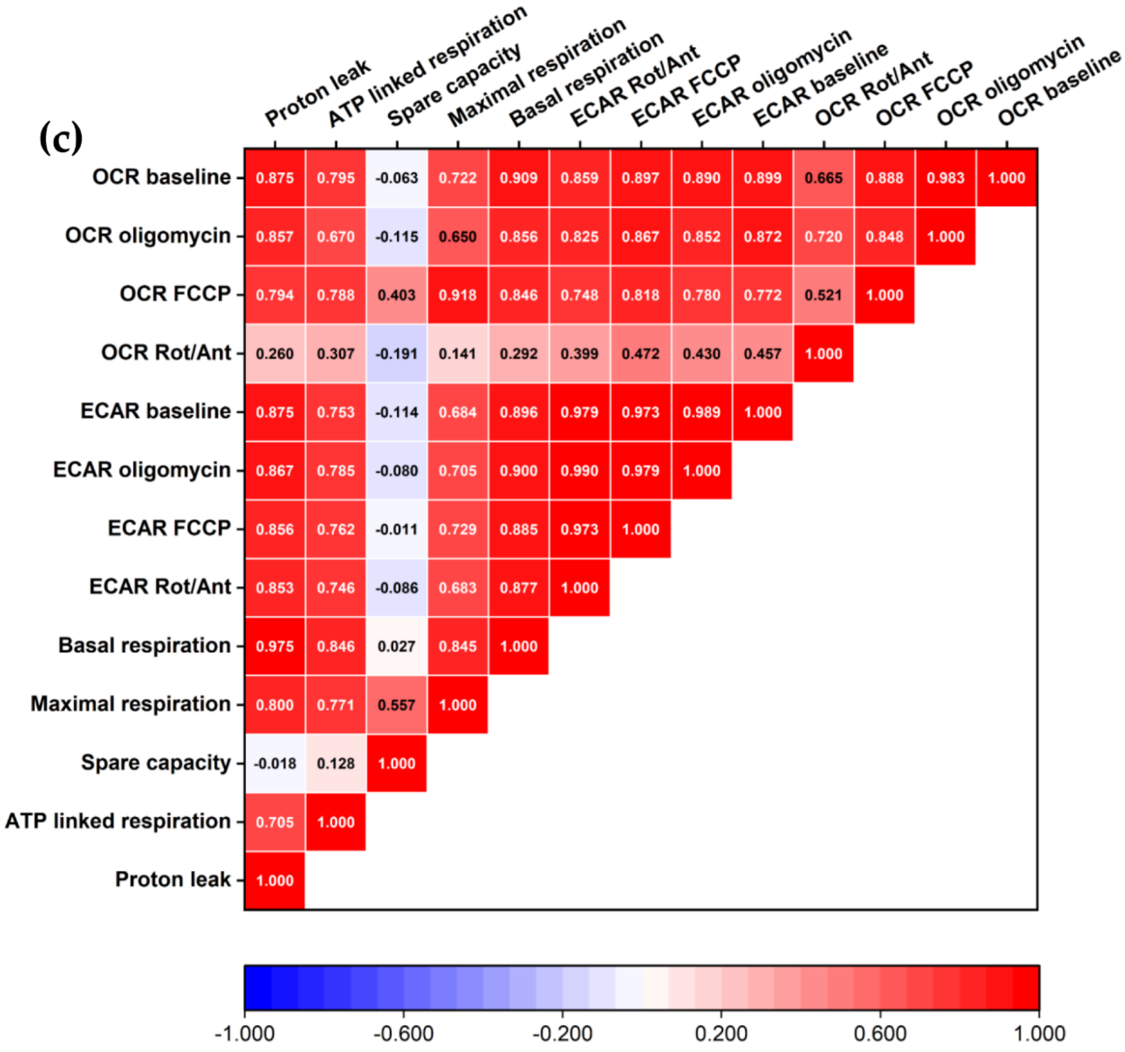

Figure S5. Impact of each respiratory parameter on PCA analyses. (**a**) PCA score plots, showing the amount of variance explained by each PCA component and the cumulative variance. (**b**) The extracted Eigenvectors define the contributes (loadings) of the original variables to the Principal Components. Values are comprised between -1 and +1. Absolute values near zero indicate that a variable contributes little to the Principal Component, whereas larger absolute values (in bold fonts) indicate significant contributions. (**c**) Correlation matrix for the original observed parameters. Values are comprised between -1 and 1, with -1 (blue) indicating a perfectly negative linear correlation between two variables, 0 (white) indicating no linear correlation, and 1 (red) indicating a perfect positive linear correlation.

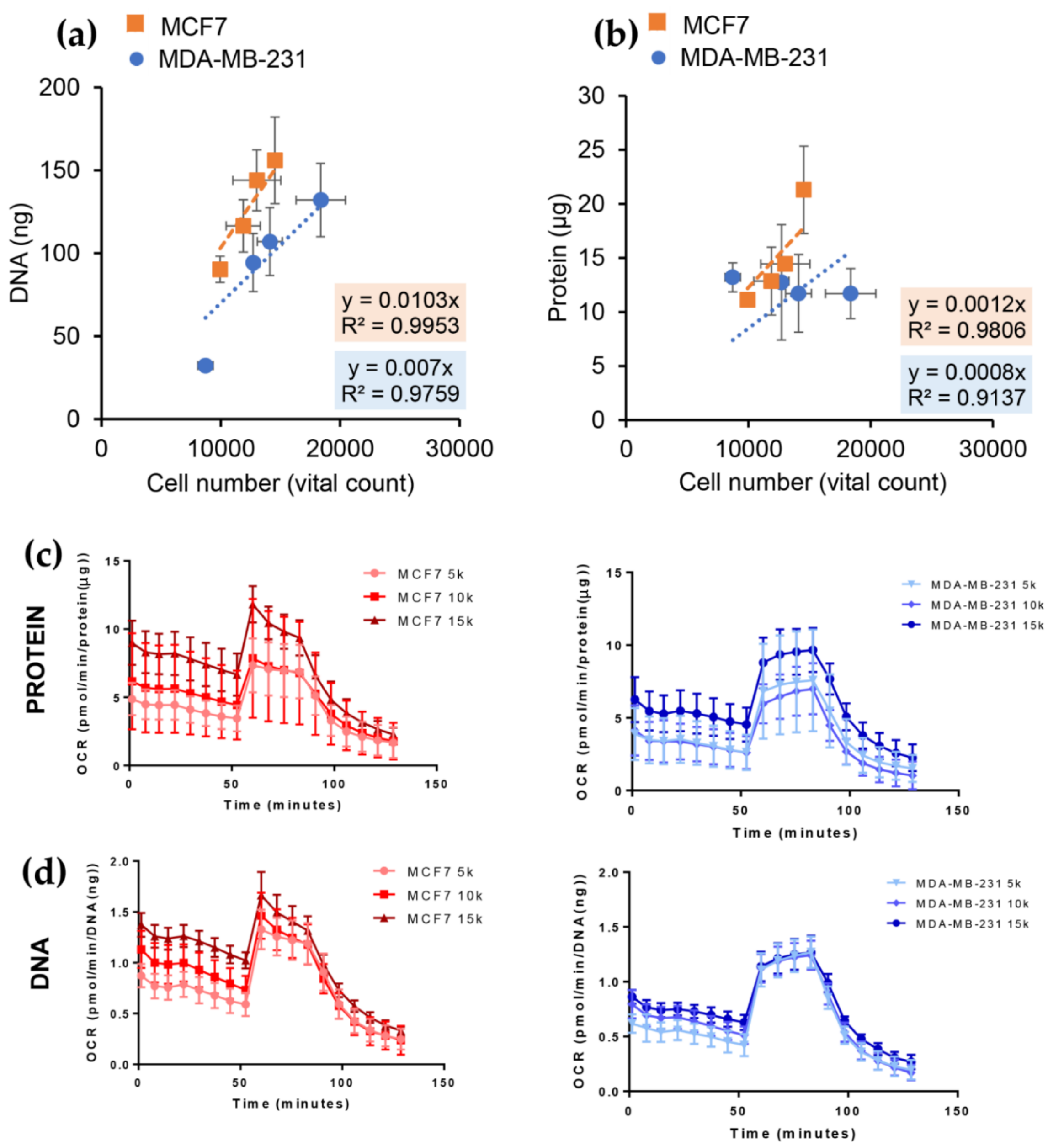

Figure S6. Other methods to normalize Seahorse parameters and relative application on Mito stress test profiles. (**a**,**b**) The curves show the relation between DNA (**a**) and protein (**b**) content and the number of vital cells in spheroids. The curves were obtained from the digestion and vital count of the cell number in groups of spheroids produced from 5,000 to 20,000 cells/well and the subsequent quantification of their protein and DNA content. (**c**,**d**) Mito stress profile was obtained testing spheroids of MCF7 and MDA-MB-231 produced by seeding a different number of cells/well (5,000 cells/well = 5k; 10,000 cells/well = 10k; and 15,000 cells/well = 15k); each point represents the OCR mean with the relative standard deviation of the indicated group of spheroids as a function of time. (**c**) OCR values normalized on the protein content of a group of spheroids directly on the XF Seahorse Microplate. (**d**) OCR values normalized on the DNA content of a group of spheroids directly on the XF Seahorse Microplate.

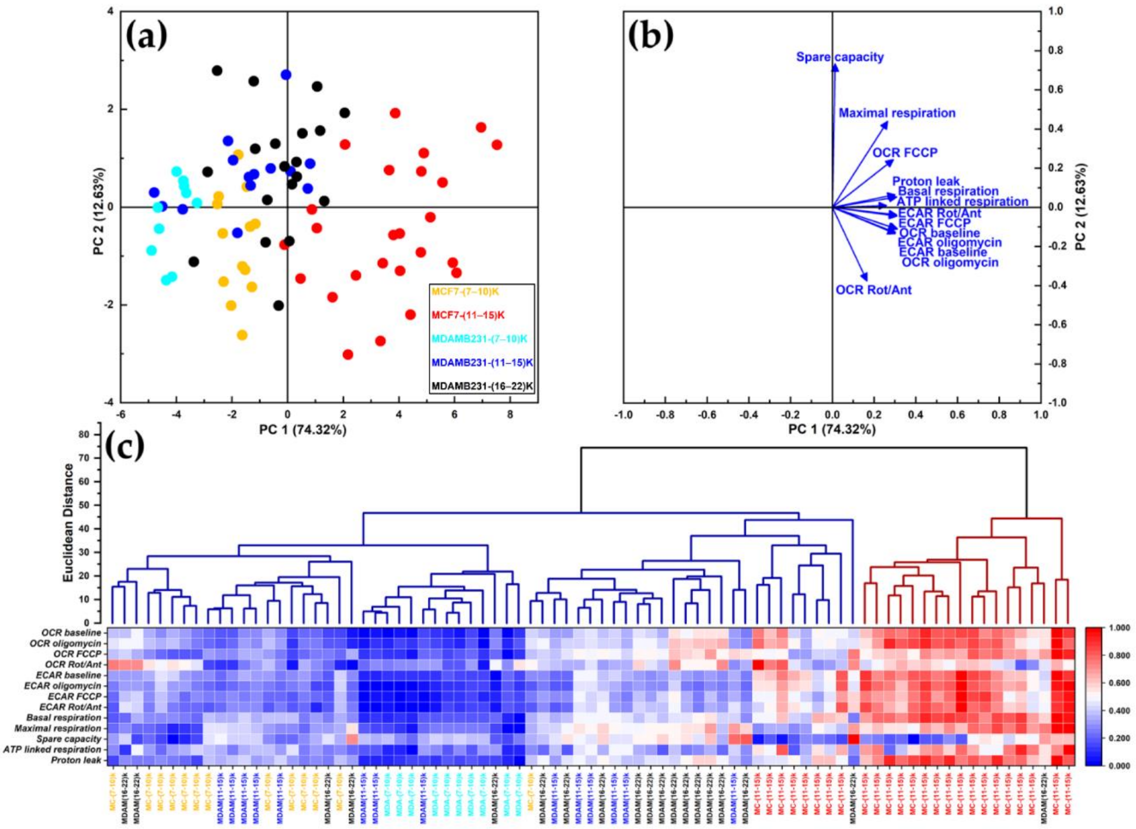

Figure S7. Multivariate statistical analysis for non-normalized metabolic parameters of MCF7 and MDA-MB-231 spheroids. (**a**) PCA scree plots, showing the amount of variance explained by each PCA component and the cumulative variance. (**b**) The extracted Eigenvectors define the contributes (loadings) of the original variables to the Principal Components. Values are comprised between -1 and +1. Absolute values near zero indicate that a variable contributes little to the Principal Component, whereas larger absolute values (in bold fonts) indicate significant contributes. (**c**) Correlation matrix for the original observed parameters. Values are comprised between -1 and 1, with -1 (blue) indicates a perfectly negative linear correlation between two variables, 0 (white) indicating no linear correlation and 1 (red) indicating a perfectly positive linear correlation.
