# Supplementary figures and images for: An Optimized Workflow for the Analysis of Metabolic Fluxes in Cancer Spheroids Using Seahorse Technology
Source: Cells. 2022 Mar 2;11(5):866. doi: 10.3390/cells11050866 (PMC8909358; doi:10.3390/cells11050866)

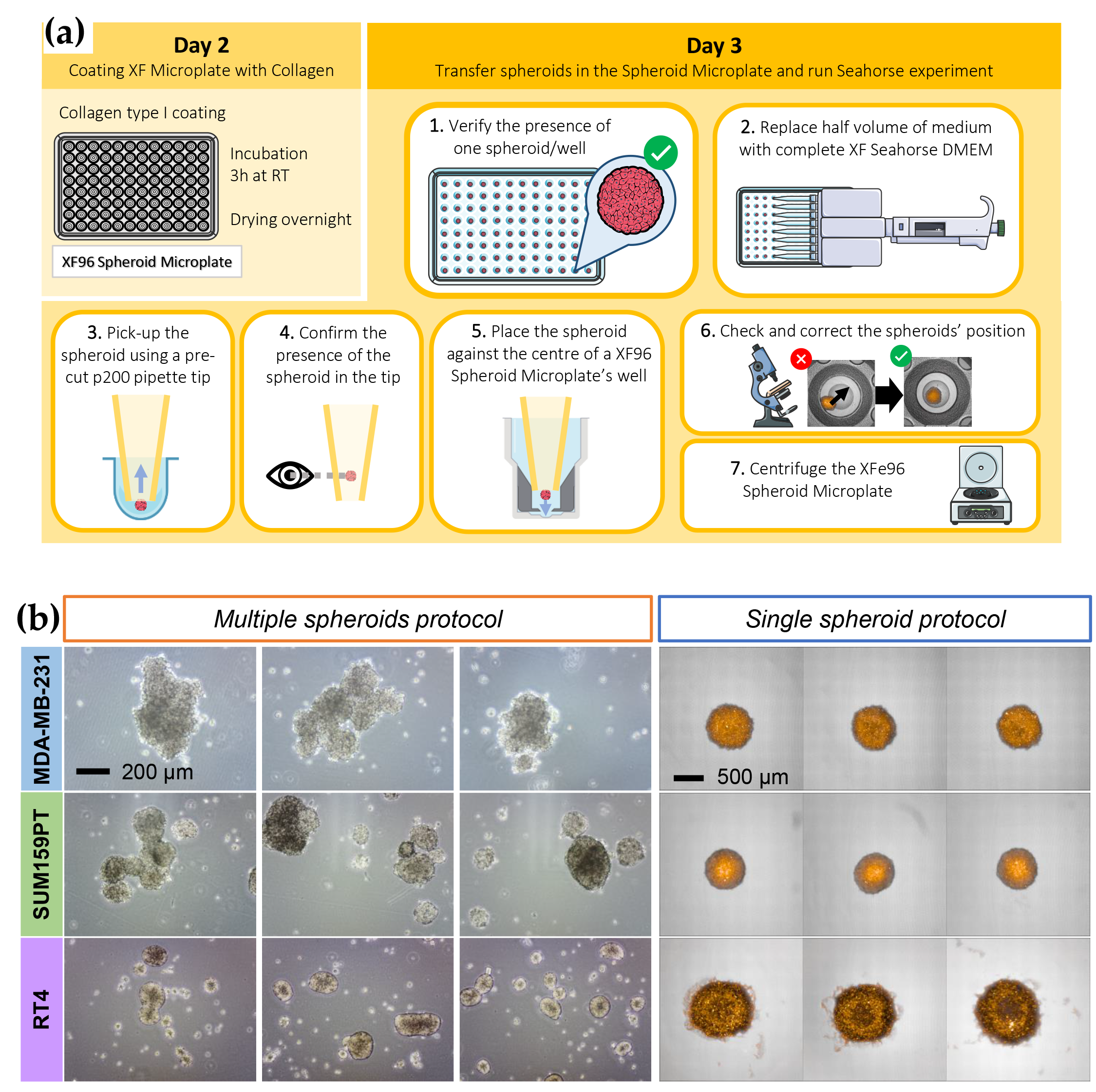

Supplement: Supplementary file 1 [file cells-11-00866-s001.zip › Figure S1.tif]

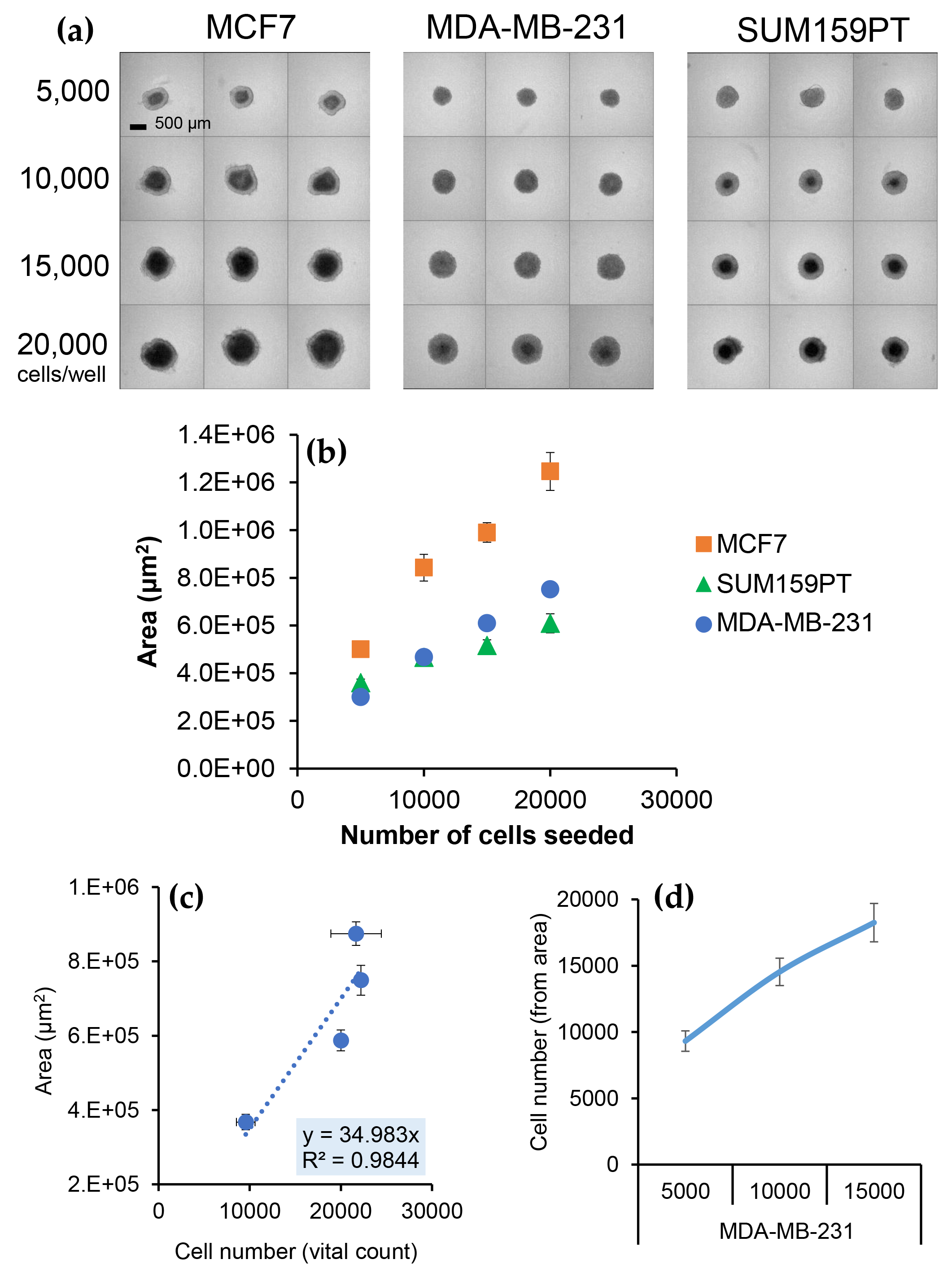

Supplement: Supplementary file 1 [file cells-11-00866-s001.zip › Figure S2.tif]

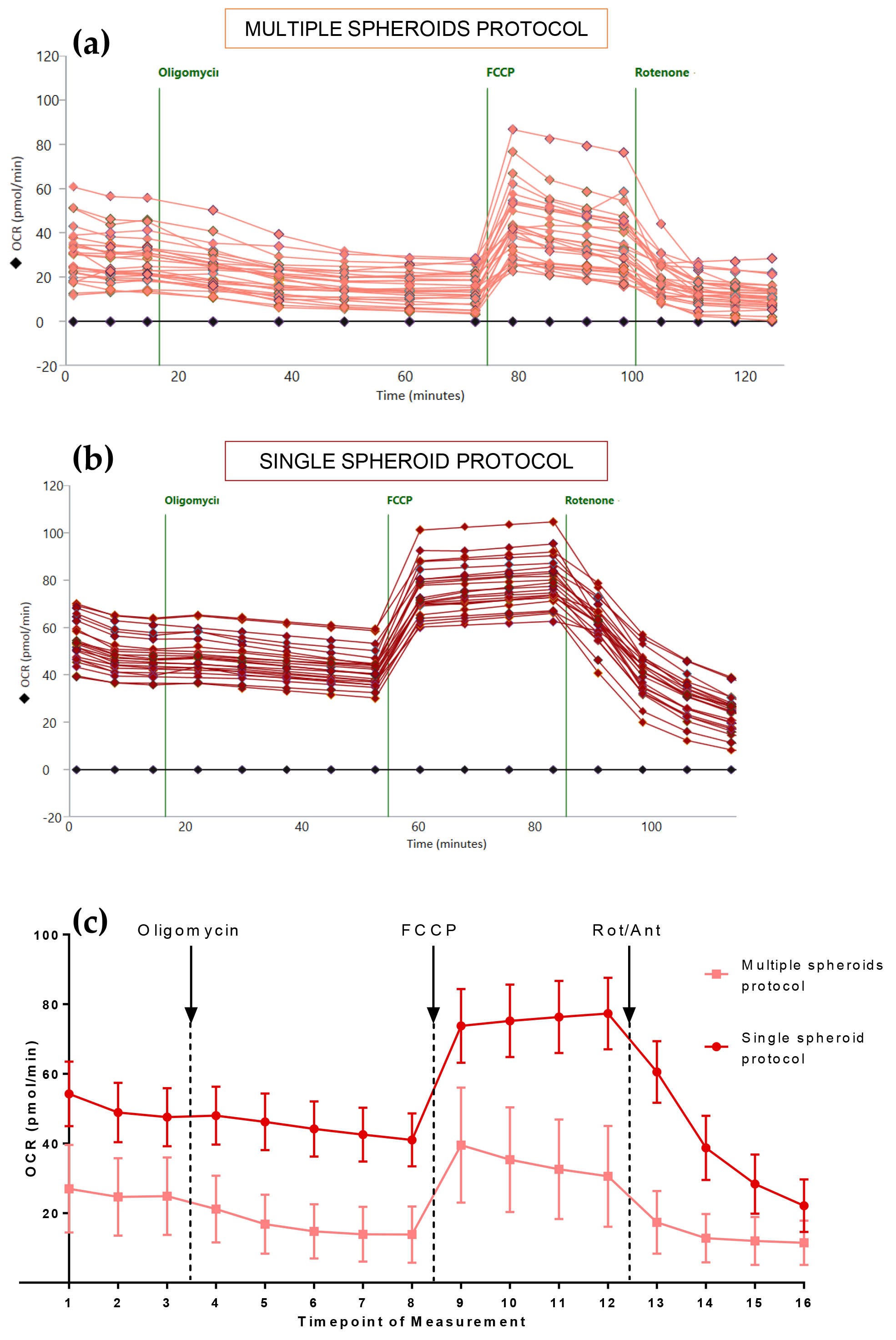

Supplement: Supplementary file 1 [file cells-11-00866-s001.zip › Figure S3.tif]

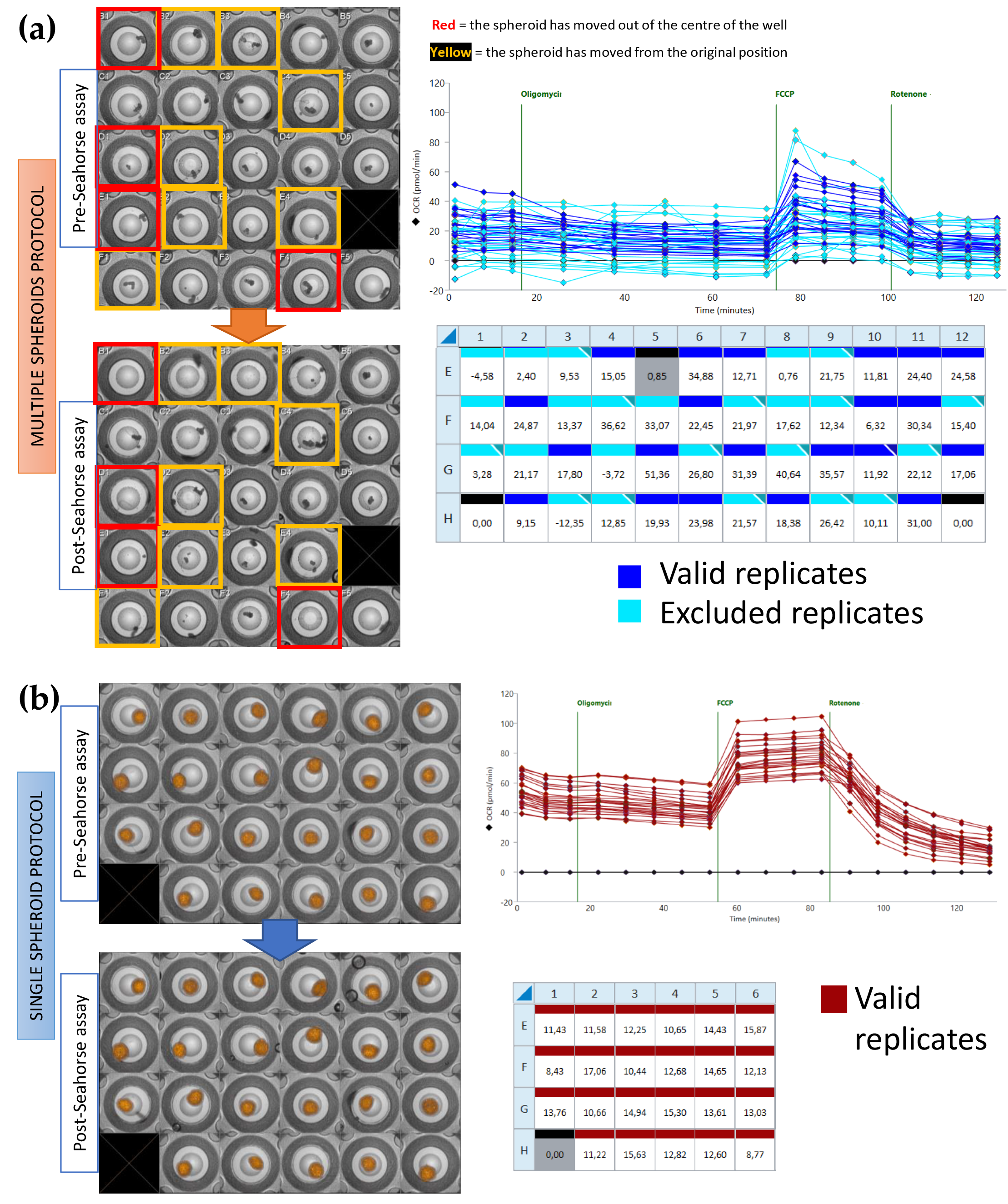

Supplement: Supplementary file 1 [file cells-11-00866-s001.zip › Figure S4.tif]

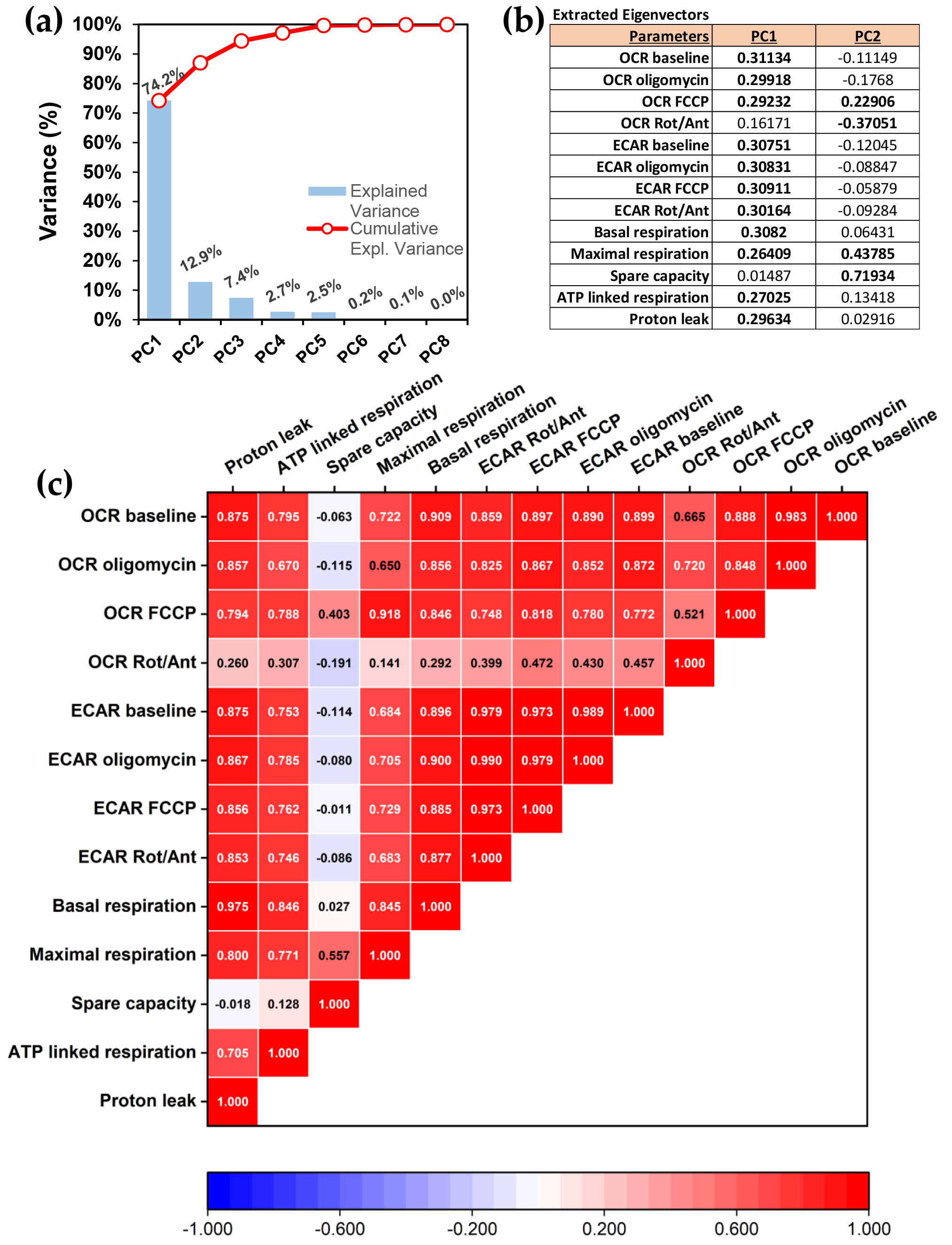

Supplement: Supplementary file 1 [file cells-11-00866-s001.zip › Figure S5.tif]

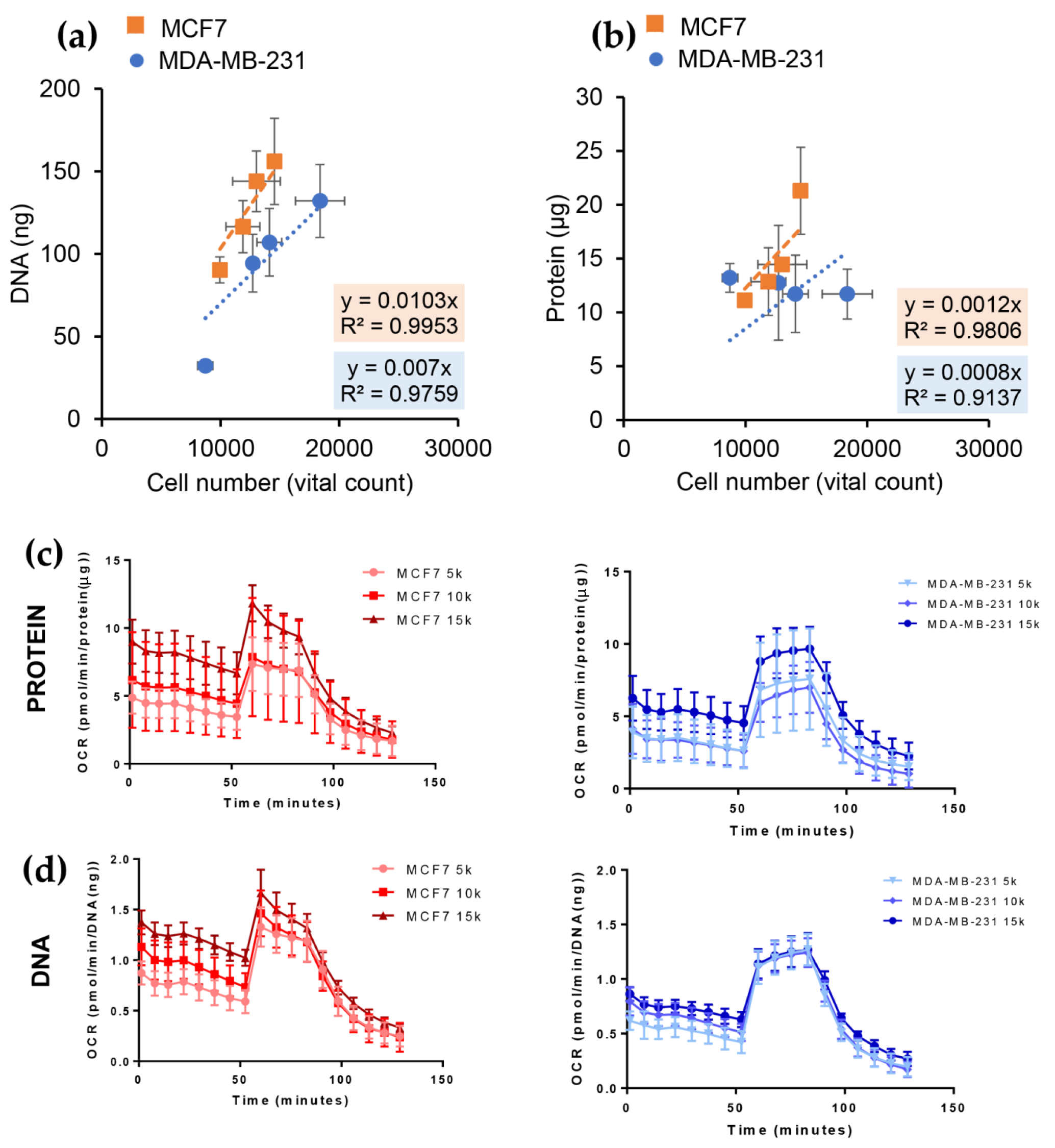

Supplement: Supplementary file 1 [file cells-11-00866-s001.zip › Figure S6.tif]

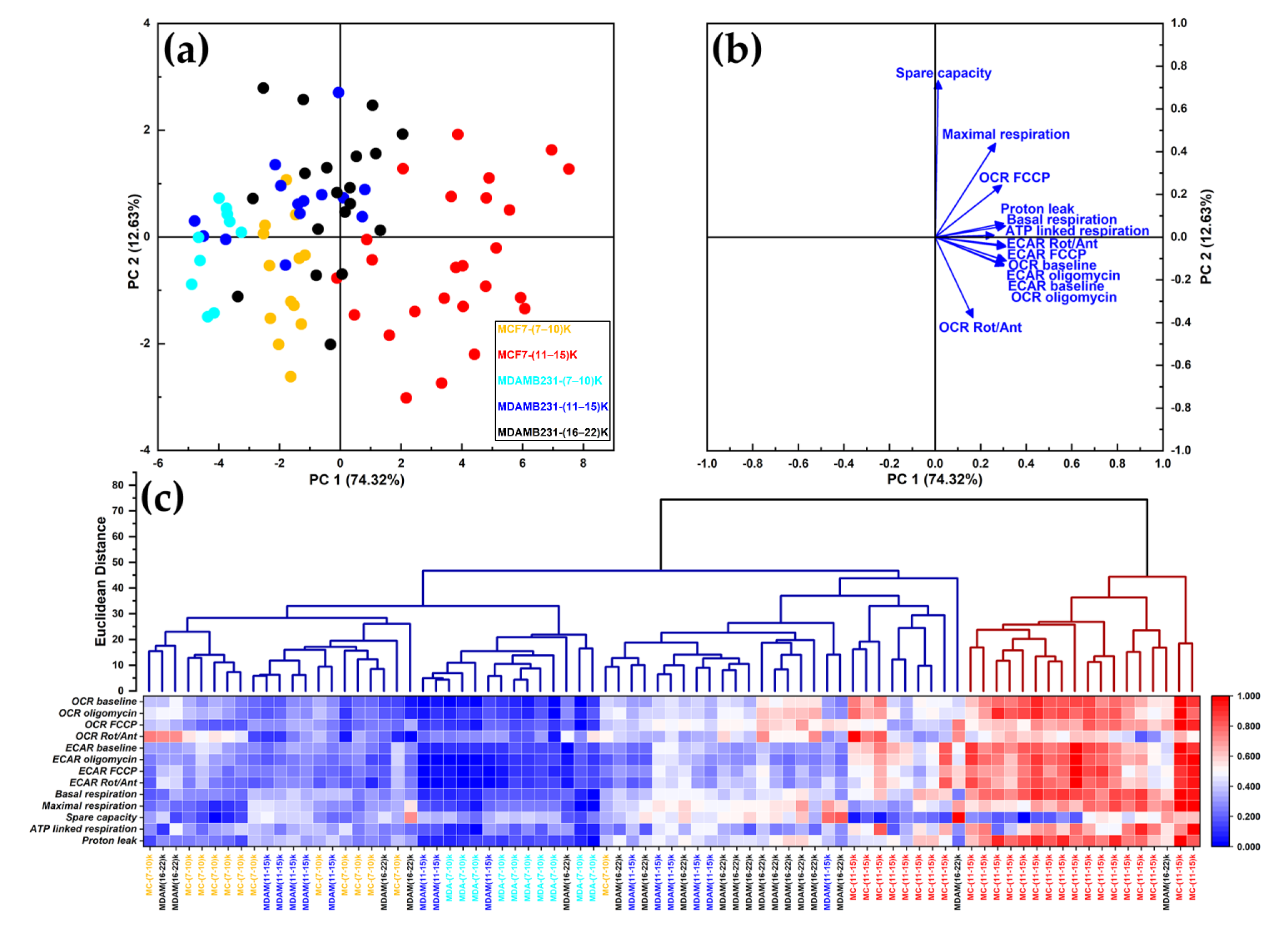

Supplement: Supplementary file 1 [file cells-11-00866-s001.zip › Figure S7.tif]
